# Incidental Extracardiac Findings and Their Characterization on Cardiac MRI

**DOI:** 10.1155/2017/2423546

**Published:** 2017-10-03

**Authors:** Matteo Gravina, Luca Pio Stoppino, Grazia Casavecchia, Angelo Pio Moffa, Roberta Vinci, Natale Daniele Brunetti, Matteo Di Biase, Luca Macarini

**Affiliations:** ^1^Radiology Department, University of Foggia, Foggia, Italy; ^2^Cardiology Department, University of Foggia, Foggia, Italy

## Abstract

**Background:**

Cardiac magnetic resonance imaging (cMRI) has recently emerged as a new noninvasive imaging modality that offers superior structural and functional assessment of the heart. cMRI benefits from a large field of view but, consequently, may capture incidental extracardiac findings (IEFs). We aimed to evaluate the frequency and significance of IEFs reported from clinically indicated cMRI scans.

**Methods:**

742 consecutive patients (402 males and 340 females) referred to the Cardiac Magnetic Resonance Center of our University Hospital between January 2015 and December 2016 for clinically indicated cMRI were retrospectively enrolled for the evaluation of IEF prevalence and relevance. The median age of the subjects was 51 years (range: 5–85 years).

**Results:**

A significant number of patients who underwent cMRI had incidental and clinically significant IEFs (2% of the population, 11.4% of cases). cMRI allowed a correct diagnosis in 116/131 cases with a diagnostic accuracy value of 88.5%.

**Conclusions:**

IEFs on cMRI are not uncommon and lesions with mild or no clinical significance represent the most frequent findings. cMRI can characterize incidental findings with high accuracy in most cases.

## 1. Background

Cardiac magnetic resonance imaging (cMRI) has recently emerged as a new noninvasive imaging modality capable of providing high-resolution images of the heart in any desired plane view, without radiation exposure. cMRI consists of several techniques that can be performed separately or in various combinations during a patient's examination. Most frequent indications for cMRI are myocarditis/cardiomyopathies, risk stratification in suspected coronary artery disease/ischemia, and assessment of myocardial viability [1, 2] and congenital heart disease.

Significant parts of neck, thorax, and upper abdomen are imaged at the time of routine clinical cMRI, particularly in the initial multislice axial and coronal images. A careful observation of the surrounding structures may therefore often identify during cMRI incidental extracardiac findings (IEFs) [3]. IEFs can represent unsuspected important diseases or benign findings, carrying several ethical, medicolegal, and financial implications [4]. Extracardiac findings during cMRI may also significantly modify clinical management of patients assessed by cMRI. Studies in literature showed different rates of prevalence of IEFs, ranging between 3 and 31% [3]. Moreover, cMRI differs from computed tomography (CT) in its use of several sequences which allows the recognizing of many differences in the appearance and conspicuity of IEFs [5]. The aim of this study was therefore to analyze retrospectively the prevalence and the nature of IEFs in a recent large series of patients referred for cMRI.

## 2. Methods

### 2.1. Patients

742 consecutive patients (402 males and 340 females) referred to the Cardiac Magnetic Resonance Center of our University Hospital (Foggia, Italy) between January 2015 and December 2016 for clinically indicated cMRI (Table 1) were enrolled retrospectively in this study for the evaluation of IEFs prevalence and relevance.

CMR examinations were all interpreted by both a radiologist and cardiologist experienced in cMRI. The diagnosis of IEF was made upon images and always included in the report.

All incidental findings discovered on cMRI were characterized by means of additional imaging techniques: ultrasound (US), computed tomography (CT), dedicated MR examination, bone scintigraphy with technetium 99m-methylene diphosphonate (99mTC-MDP), and positron emission tomography-computed tomography (PET-CT).

IEFs were classified into three categories: (1) findings with mild or no clinical significance, (2) findings with possible clinical significance, and (3) clinically significant findings. The overall prevalence and the sites of extracardiac findings were evaluated and reported.

Written informed consent was obtained from all patients; the study was held according to the principles of the Declaration of Helsinki.

### 2.2. CMR Protocol

CMR protocols were based on standardized protocols recommended by the Society for Cardiovascular Magnetic Resonance (SCMR) and the European Society of Cardiology (ESC) Working Group EuroCMR, respectively [6].

CMR was performed using a 1.5 T magnet (Achieva, Philips Healthcare, Best, Netherlands) with a cardiac phased-array receiver coil with cardiac gating. The standard protocol included multiplane steady-state free precession (SSFP) localizers, transversal T1-TSE black blood, sequences cine steady-state free precession (SSFP) oriented 2, 3, 4 chamber and short axis for the study of the kinetics of the right and left ventricles, T2 short-tau inversion recovery (STIR) black blood for the study of myocardial oedema in short axis and 4 chamber, dynamic sequence T1-TFE in short axis, Phase-contrast to study valvular flow, Phase Sensitive Inversion Recovery (PSIR) sequences for the study of late gadolinium enhancement (LGE) performed after 10–15 minutes after intravenous administration of gadolinium (0.1 mmol/kg). Field of view (FOV) of CMR sequences is the determining factor for the highlight of the exhibits around the heart and it is standard according to the international protocols.

### 2.3. Statistical Analysis

Continuous variables were reported as mean ± standard deviation or median and interquartile range, dichotomic as percentages.

## 3. Results

The median age of the subjects was 51 years (range: 5–85 years). Incidental findings were found in 109/742 (14.7%) of examined patients for a total of 131 IEFs; of these, 52 (40%) were intrathoracic and the remaining 79 (60%) were located in upper abdomen. 15 out of 131 incidental findings (11%) were confirmed to be clinically significant, while in the remaining 116 findings 87 were considered to be of mild or no clinical significance (66%); 29 were considered to be of possible clinical significance (22%) based on patient's clinical condition (Table 2).

Of the 131 collateral findings, 15 in 15 patients (prevalence: 2%) were classified as significant and deserving further diagnostic work-up: mediastinal lymphadenopathy/mass (6/15), lung nodule/mass (3/15), aortic coarctation (2/15), breast nodule (1/15), complex renal cyst (1/15), hepatic mass (1/15), and solid renal mass (1/15). Findings with possible clinical significance (29/131) were present in 22 patients (prevalence: 3%): pleural effusion/thickening (9/29), thyroid goitre (6/29), gallbladder lithiasis (5/29), airspace disease (4/29), splenomegaly (2/29), hydronephrosis (2/29), and adrenal nodule (1/29). IEFs with mild or no clinical significance were found in 72 patients (prevalence: 9.7%): simple renal cyst (31/87), hepatic cyst (24/87), hiatal hernia (8/87), hepatic haemangioma (8/87), thyroid nodule < 1 cm (7/87), bone haemangioma (3/87), paraspinal cyst (2/87), and splenic cyst (1/87) (Table 3).

The most common site of IEFs' localization was the kidney (35/131, 26.7%), followed by liver (33/131, 25.2%), lung (19/131, 14.5%), and thyroid (13/131, 9.9%). The lesions found in spleen, pleura, and gallbladder resulted to be with mild or possible clinical significance in all cases and no further diagnostic work-up was deemed necessary. Lung lesions resulted to be malignant in all cases including metastases in 2/3 and lung cancer in 1/3 of cases. Only one of 33 (3%) focal liver lesions resulted to be a metastatic hepatic mass. Two out of 35 (6.7%) kidney lesions resulted to be malignant including 1 case of complex renal cyst and 1 case of renal cell cancer. In two other cases of kidney lesions (6.7%), we observed unilateral hydronephrosis caused by kidney stones, as confirmed by additional imaging. Comparing MR findings with the additional definitive imaging tools, cMRI allowed a correct diagnosis in 116/131 cases with a diagnostic accuracy value of 88.5%. In particular, a lung mass was misdiagnosed as a cancer by cMRI, while it was shown to be a pulmonary atelectasis on chest CT examination.

A new/previously unknown diagnosis was made in 74% of cases with IEFs. The most informative sequences for IEFs were surveys (Balanced-TFE), that is, the initial locating sequences in the three planes of space which allow a global view of neck, chest, and abdomen, 90% of IEFs. Further relevant sequences for details were morphological sequences T1-TSE and T1-Fat Sat before and after contrast imaging (5%) and T2-STIR (5%).

## 4. Discussion

cMRI is a highly reproducible tool to assess myocardial morphology as well as global and regional heart function. It also provides relevant information regarding tissue characteristics such as viability, myocardial perfusion, storage diseases, and inflammation. cMRI is thus increasingly used in daily practice [7].

In cMRI examinations, a careful assessment of noncardiac structures may also detect relevant noncardiac diseases. The wide FOV used to perform axial/coronal SSFP and BB-FSE sequences at the beginning of the CMR examination allows exploring surrounding cardiac structures including the lungs, upper abdomen, and thoracic spine. This enables detecting possible IEFs that could be clinically significant or require further diagnostic work-up [8]. Few studies are reported in literature concerning the prevalence and the nature of IEFs on cMRI; their comparison is difficult because of different study design (i.e., cohort studied, "clinical setting," sequences applied, and reading session format). Indeed, IEFs based on CMR reports' review were reported by some authors [9], while others, as in the current study, performed an extensive image analysis [10]. In our series, IEFs were encountered in 109 (14.7%) of the 742 examined patients. The prevalence of IEFs in our study population is slightly higher than that previously observed: about twofold greater than Chan et al. [11] in 1534 consecutive clinically indicated cMRI studies (15% versus 7.6%) and almost threefold greater than Ulyte et al. [12] (15% versus 5.3%) in a review of 4165 cMRI reports. Sohns et al. [10] reported in 234 cMRI studies a slightly higher rate of extracardiac findings (26% of 854 patients), almost comparable with the prevalence in Irwin et al.'s work [9] (21.4% of 714 patients). A recent systematic review and meta-analysis of 12 studies including data from 7,062 patients demonstrated pooled prevalence of incidental extracardiac findings of 35% [13].

**Figure 1:**
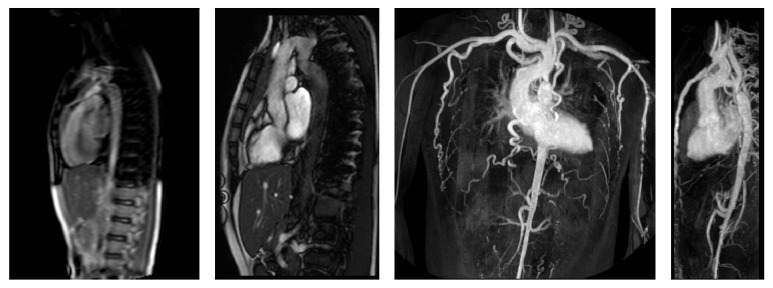
Sagittal localizers, SSFP 2 chamber, and 3D-CE-MRA images of aortic coarctation.

**Figure 2:**
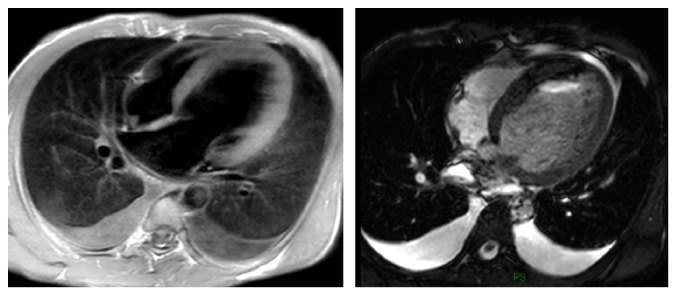
Bilateral pleural effusion.

**Figure 3:**
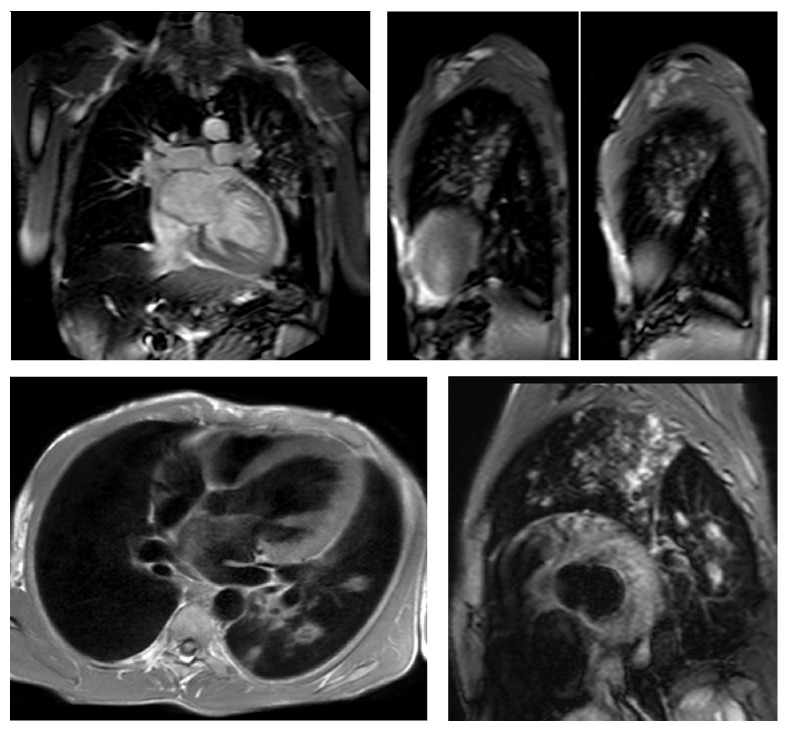
Coronal and sagittal localizer SSFP, Transverse T1-TSE, and short axis STIR-T2 images showing pulmonary irregular opacities in the upper lobe and apical segment of the lower lobe of the left lung compatible with a diagnosis of secondary tuberculosis.

**Figure 4:**
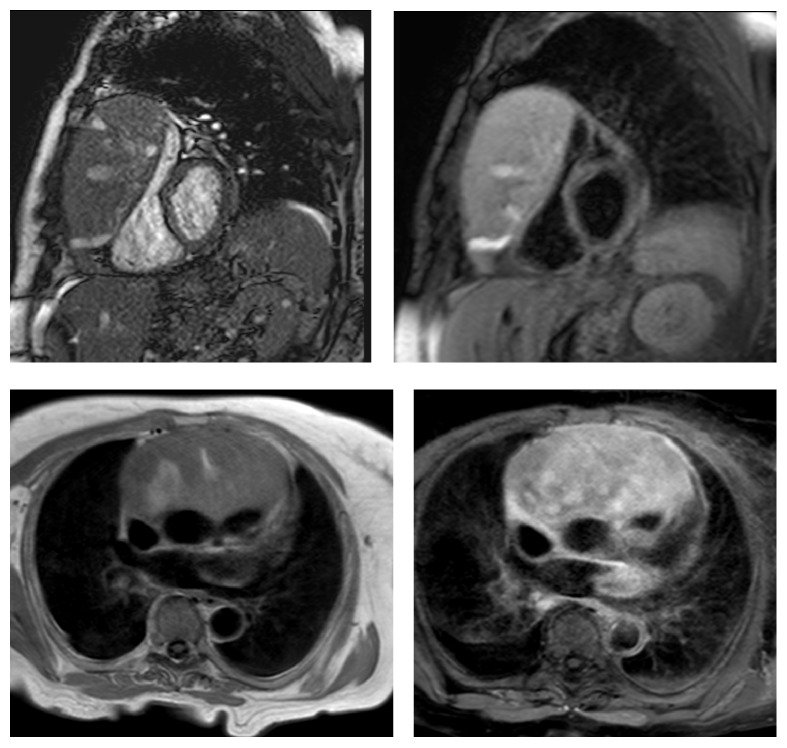
Cine-SSFP short axis and T2-STIR short axis and T1-TSE without gadolinium and T1-TSE-SPIR images with gadolinium showing a voluminous mass in the anterior mediastinum compressing right heart chambers.

**Figure 5:**
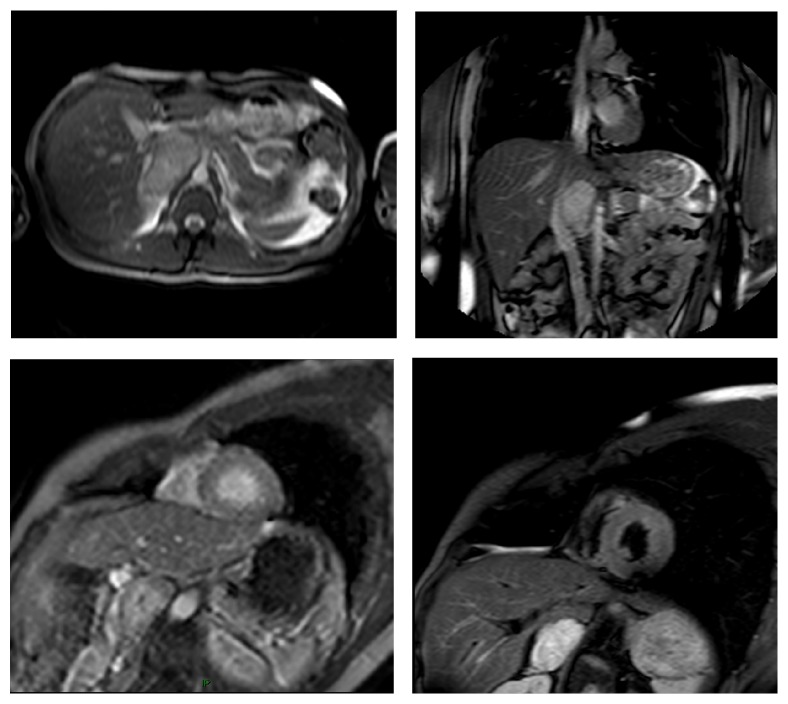
Axial and coronal localizer SSFP, short axis perfusion, and STIR-T2 short axis images showing an adrenal mass compatible with a diagnosis of pheochromocytoma confirmed at histology.

In the present study, images (and not cMRI reports) were analyzed in order to assess the incidence of IEFs. According to Klysik et al. [14], in our study, IEFs were classified into three categories: findings with mild or no clinical significance, findings with possible clinical significance, and clinically significant findings. Most of findings with mild or no clinical significance could generally be ignored without consequence for the patient's outcome. Findings with possible clinical significance may require additional imaging depending on patients' clinical condition or due to their nondedicated imaging. Finally, the clinically significant findings need immediate evaluation or treatment and further diagnostic work-up should be mandatory. In our study, we found two cases of aortic coarctation; sequences dedicated cine-MRI type allowed a detailed study of the anomalies (Figure 1); further CT scan examinations were therefore considered unnecessary. Vascular abnormalities (such as a case of abnormal pulmonary venous return undiagnosed earlier), mild and severe pleural effusions (Figure 2), consolidative pulmonary parenchymal phenomena (Figure 3), and both benign and malignant pulmonary nodules were found (Figure 4). Extracardiac mediastinal masses were also revealed with an accurate analysis of the relationship with the cardiac structures and of mediastinal adenopathy. A case of Takotsubo cardiomyopathy is noteworthy with an adrenal mass characterized by an intense enhancement at first pass; a pheochromocytoma was confirmed at following histological examination [15, 16] (Figure 5).

**Figure 6:**
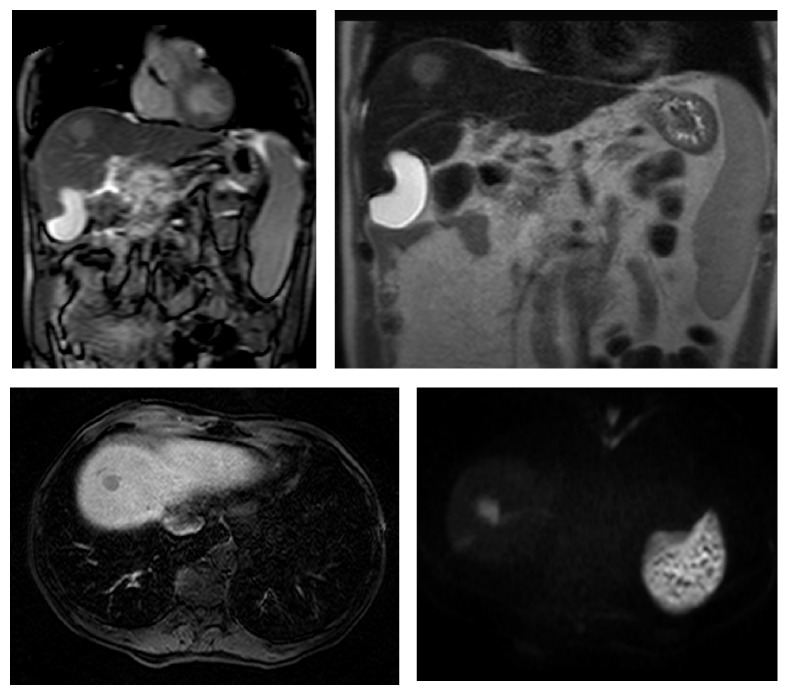
Coronal localizer SSFP, coronal T2-TSE, and Transverse Thrive images with gadolinium and diffusion weighted images of a case of hemochromatosis with a liver nodular hepatocellular carcinoma.

In the present study, clinically significant extracardiac findings were observed in 2% of the population (11.4% of cases), which is consistent with previous studies that reported similar prevalence for “major” IEFs during cMRI (range: 1–27%) [10]. The most frequent clinically significant IEF was mediastinal lymphadenopathy/mass (defined as >1 cm in the short axis), which was encountered in 6 patients. However, the majority of IEFs were less important and were associated with a benign diagnosis. In fact, in 65% of cases, extracardiac lesions detected on CMR were benign. The most common site of IEFs' localization was the kidney (26.7%), followed by the liver (25.2%) (Figure 6), the lung (14.5%), and thyroid (9.9%). The results of this study demonstrate that a significant number of patients who underwent cMRI may present IEFs. However, a small percentage of these occasional findings actually have a clinical relevance and deserve further diagnostic investigation.

cMRI may be extremely useful in characterizing these incidental findings, with an excellent diagnostic accuracy (88.5%), although further imaging techniques are often necessary to precisely define incidental findings. In particular, CT and fluorodeoxyglucose PET-CT (FDG PET-CT) scan are the first choice to characterize and discriminate the nature of incidental lung parenchymal lesions. Ultrasound (US) or dedicated MRI may be used to further evaluate abdominal and breast lesions. Finally, in all cases of incidental bony lesions, MR with dedicated sequences or bone scintigraphy may improve the characterization of IEFs.

## 5. Conclusions

A significant number of IEFs can be detected during cMRI, with high accuracy. It is therefore extremely important that whoever reports cMRI should be able to properly assess normal and abnormal thorax and superior abdominal findings. IEFs should be searched, for potentially modifying the clinical management of patients with such findings.

## Figures and Tables

**Table 1:** Clinical indications for cardiac magnetic resonance imaging studies (*n* = 742).

Indication for CMR	Number of cases
Myocarditis/cardiomyopathies	356 (47.9%)
Coronary artery assessment	168 (22.6%)
Cardiac masses	60 (8%)
Valvular disease	56 (7.5%)
Congenital heart disease	42 (5.6%)
Pericardial disease	31 (4.2%)
Myocardial viability	26 (3.5%)
Others	3 (0.4%)

**Table 2:** Prevalence of incidental extracardiac findings at cardiac magnetic resonance imaging.

Category	Number of cases	Patients	Prevalence
Mild or no clinical significance	87	72	9.7%
Possible clinical significance	29	22	3%
Clinically significant	15	15	2%
Total	131	109	14.7%

**Table 3:** Frequency of incidental extracardiac findings at cardiac magnetic resonance imaging.

Mild or no clinical significance		Possible clinical significance		Clinically significant	
Simple renal cyst	31	Pleural effusion	9	Mediastinal lymphadenopathy/mass	6
Hepatic cyst	24	Thyroid goitre	6	Lung nodule/mass	3
Hiatal hernia	8	Gallbladder lithiasis	5	Aortic coarctation	2
Liver haemangioma	8	Airspace disease	4	Breast nodule	1
Thyroid nodule < 1 cm	7	Splenomegaly	2	Complex renal cyst	1
Pleural thickening	3	Hydronephrosis	2	Hepatic mass	1
Paraspinal cyst	2	Adrenal nodule	1	Solid renal mass	1
Splenic cyst	1				
*Total*	87		29		15
Recommendation					
*No further work-up is necessary*		*Further work-up is recommended depending on specific clinical scenario*		*Further diagnostic work-up is mandatory *	
